# Supplementary figures and images for: Unraveling the association between gut microbiota and chemotherapy efficacy: a two-sample Mendelian randomization study
Source: Microbiol Spectr. 2024 Jul 11;12(8):e03948-23. doi: 10.1128/spectrum.03948-23 (PMC11302730; doi:10.1128/spectrum.03948-23)

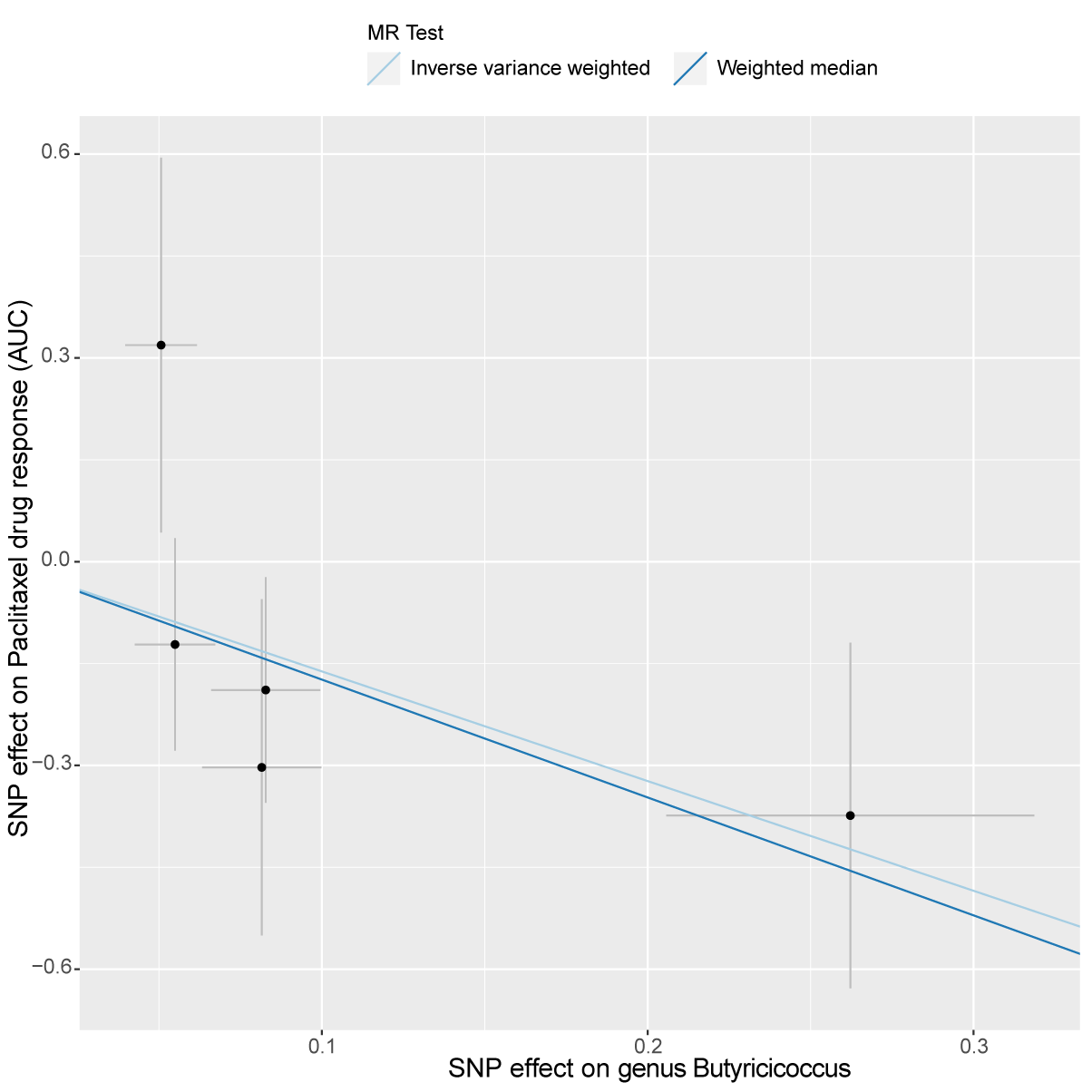

Supplement: Fig. S1 — Scatter plot. [file spectrum.03948-23-s0001.tif]

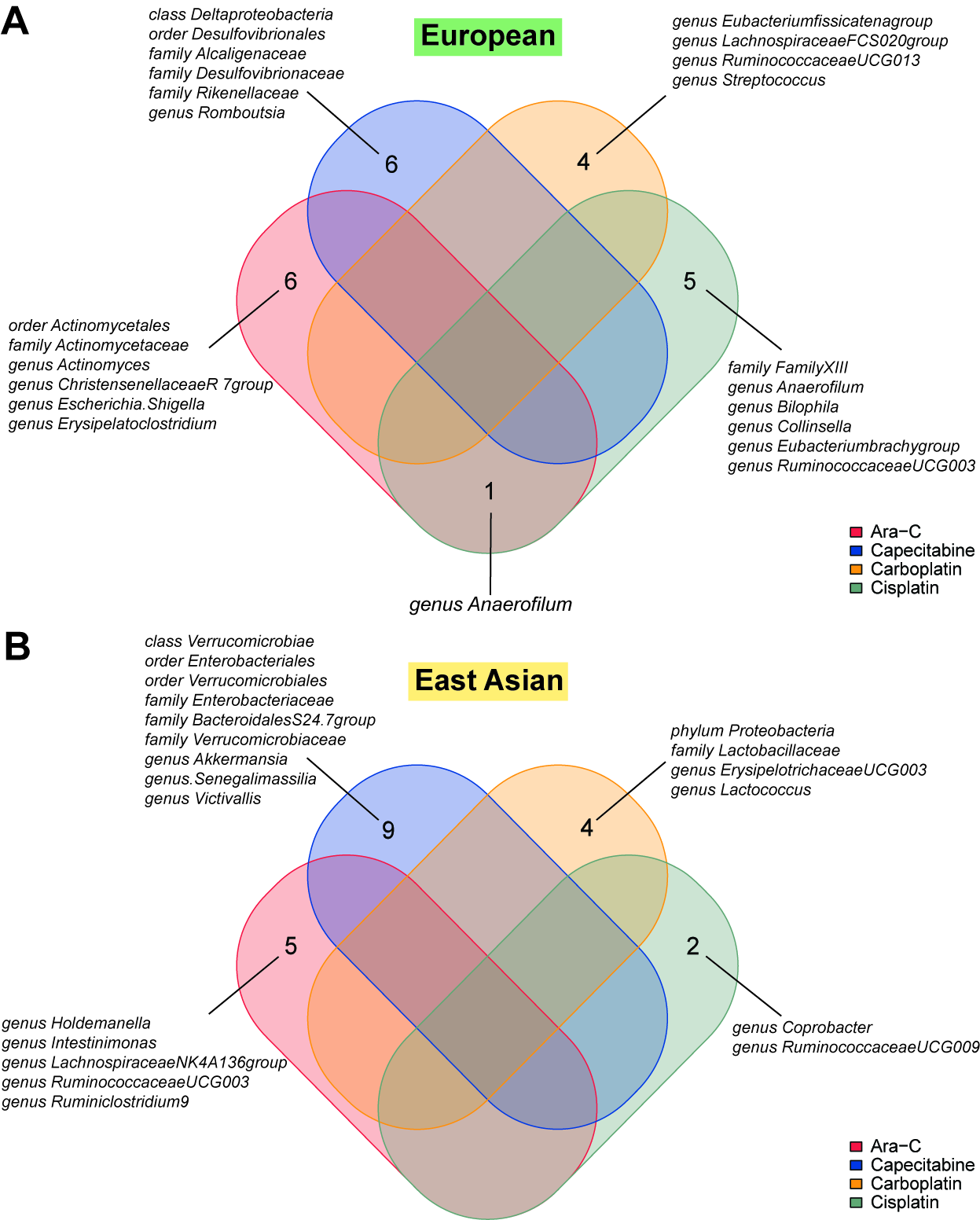

Supplement: Fig. S2 — Venn diagrams. [file spectrum.03948-23-s0002.tif]

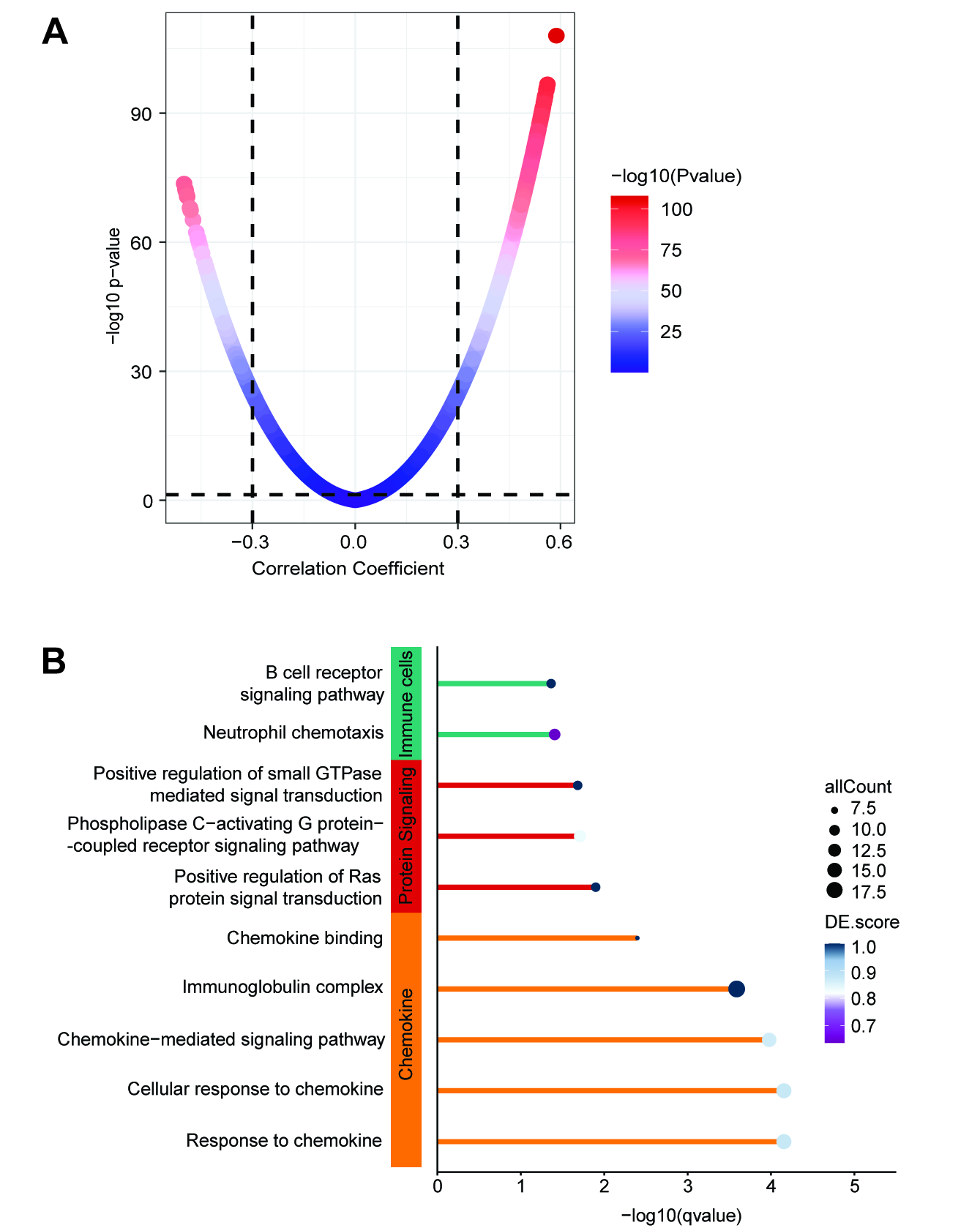

Supplement: Fig. S3 — Correlation analysis of six microbial taxa abundance and gene expression levels. [file spectrum.03948-23-s0003.tif]
