# Supplementary material for: Unraveling the association between gut microbiota and chemotherapy efficacy: a two-sample Mendelian randomization study
Source: Microbiol Spectr. 2024 Jul 11;12(8):e03948-23. doi: 10.1128/spectrum.03948-23 (PMC11302730; doi:10.1128/spectrum.03948-23)
Supplement: Supplemental material — Captions for figure and tables. [file spectrum.03948-23-s0004.docx]

**Supplementary Fig S1** Scatter plot illustrating the association of IVs related to genus *Butyricicoccus* with paclitaxel drug response in SubSaharan African population. **Abbreviations**: IVs, instrumental variables; MR, mendelian randomization; SNP, single nucleotide polymorphism.

**Supplementary Fig S2** Venn diagrams showing the causal relationships between gut microbiota and different chemotherapeutic drug responses in European (A) and East Asian populations (B).

**Supplementary Fig S3** Correlation analysis of six microbial taxa abundance (A) and gene expression levels in tumor tissues and gene pathway analysis (B).

**Supplementary Table S1** Details of 67 IVs related to 7 gut microbial taxa significantly associated with chemotherapy efficacy.

**Supplementary Table S2** Comprehensive results of MR analyses elucidating the association between 196 gut microbial taxa and chemotherapy efficacy.

**Supplementary Table S3** Comprehensive results of MR analyses elucidating the association between 6 gut microbial taxa and various chemotherapy drug response across diverse ethnicities.

**Supplementary Table S4** Correlation between the abundance of six microbial taxa in tumor tissues and gene expression levels.
